# Autumnal migration patterns of hoverflies (Diptera: Syrphidae): interannual variability in timing and sex ratio

**DOI:** 10.7717/peerj.14393

**Published:** 2022-12-09

**Authors:** Antonín Hlaváček, Radek K. Lučan, Jiří Hadrava

**Affiliations:** Faculty of Science, Department of Zoology, Charles University Prague, Prague, Czech Republic

**Keywords:** Biogeography, Insect, Phenology, Flower flies, Sex ratio, Migration

## Abstract

**Background:**

The migration of hoverflies (Diptera: Syrphidae) is a well-known phenomenon, with growing interest due to the ecosystem services provided by migrants. However, we still lack fundamental data on species composition, timing of migration, or sex ratio of migrants. To address this gap, we focused on the southward autumnal migration of hoverflies through central Europe.

**Methods:**

To recognize migrating individuals from resident ones, we used a pair of one-side-blocked Malaise traps, exposed in a mountain pass in the Jeseníky mountains, Czech Republic, where a mass migration of hoverflies takes place annually. Traps were set for 4 years, from August to October.

**Results:**

In total, we recorded 31 species of migrating hoverflies. The timing of migration differed between the years, taking place from the beginning of September to the end of October. Differences in phenology were observed in the four most common migrant species, where larger species seemed to migrate earlier or at the same time compared to the smaller ones. The sex ratio was strongly asymmetrical in most common species *Episyrphus balteatus*, *Eupeodes corollae*, and *Sphaerophoria scripta*, and varied between years for each species. Weather conditions strongly influenced the migration intensity at ground-level: hoverflies migrate mainly during days with south wind, high temperature, high atmospheric pressure, and low precipitation.

## Introduction

Autumnal migration of insects is a well-known phenomenon around the world (Williams, 1957; Chapman, Reynolds & Wilson, 2015; Wotton et al., 2019). Massive movements of insect biomass provide pollination services, crop protection, and transport of nutrients (Svensson & Janzon, 1984; Wotton et al., 2019, Gilbert, 2019). Although a large number of insect species are known to exhibit migratory behaviour, the natural history of most migratory species remains elusive, except ‘model organisms’ of migratory research such as monarch butterfly *Danaus plexippus* (Brower, 1961; Nail, Drizd & Voorhies, 2019; Tracy et al., 2019; Chowdhury et al., 2021) or migratory locust, *Locusta migratoria* (Uvarov, 1921; Tu et al., 2020; Peng et al., 2020). The importance of hoverfly migration was overlooked for a long time.

The migration of hoverflies was first reported in 1864, when massive swarms of *Scaeva pyrastri* L. 1758 were observed on beaches of Dorsetshire (Symes, 1864). Dozens of observations of autumnal mass migrations of hoverflies were given later (*Palearctic*: Saunt, 1945; Lack, 1951; George, 1960; Speight, 1996; Jensen, 2001; Kehlmaier, 2002; Nielsen, 2009; Nielsen et al., 2010; Massy et al., 2021; *Nearctic*: Shannon, 1926; Menz, Brown & Wotton, 2019; *Australia*: Kelly, 2017; Finch & Cook, 2020; *Oriental*: Westmacott & Williams, 1954), providing an important baseline for further research.

Beside those reports, which were usually based on observations on a single locality and in a single year, most studies focused on ecosystem services provided by migrating hoverflies (Gilbert, 2019; Wotton et al., 2019; Gao et al., 2020) or were dealing with the navigation of migrants (Gao et al., 2020; Massy et al., 2021; Hawkes et al., 2022a). Only a few studies systematically documented the diversity of migrants (Aubert, Aubert & Goelding, 1976; Gatter et al., 2020, Hawkes et al., 2022b) or provided more detailed information about the migrants *e.g.*, sex ratio as in Svensson & Janzon (1984), Hondelmann (2007), or Martens (2020).

Furthermore, most studies have focused only on economically important species (*Eupeodes corollae* (F., 1794): Svensson & Janzon, 1984; Speight, 1996. *Episyrphus balteatus* (De Geer, 1776): Hondelmann, 2007; Dällenbach et al., 2018), thus the migratory behaviour of other species remains unknown, although a substantial part of hoverfly diversity is known to exhibit migration behaviour (Speight, 2020). Also, the timing of migration and its drivers are only poorly understood.

Migratory insects select for favourable weather conditions (Taylor, 1974; Gao et al., 2020; Massy et al., 2021), including temperature, barometric pressure, and mainly the active selection of suitable wind direction (Brattström et al., 2008; Gao et al., 2020; Knoblauch, Thoma & Menz, 2021). Autumnal migrants appear to select between high-altitude tailwind and low-altitude flight when facing headwinds, flying on the flight boundary layer (Taylor, 1974; Massy et al., 2021). In recent years, new methods such as vertical entomological radars discovered huge movement of biomass and so revealed the ecological significance of the migration (Drake et al., 1981; Chapman, Reynolds & Smith, 2003; Drake & Reynolds, 2012; Wotton et al., 2019).

In this study, we report our findings on the autumn migration of hoverflies, observed during the autumns of 2018, 2019, 2020, and 2021 on the Červenohorské sedlo mountain pass in the Jeseníky Mountains, Czech Republic. The goals of this study were (1) to investigate the diversity, migration timing, and sex ratio of European migratory species of hoverflies, (2) to examine the interannual variability in hoverflies migration patterns, and (3) to investigate influence of weather conditions on hoverfly migration at the ground level through central Europe.

## Materials and Methods

### Study area and sampling

**Figure 1:**
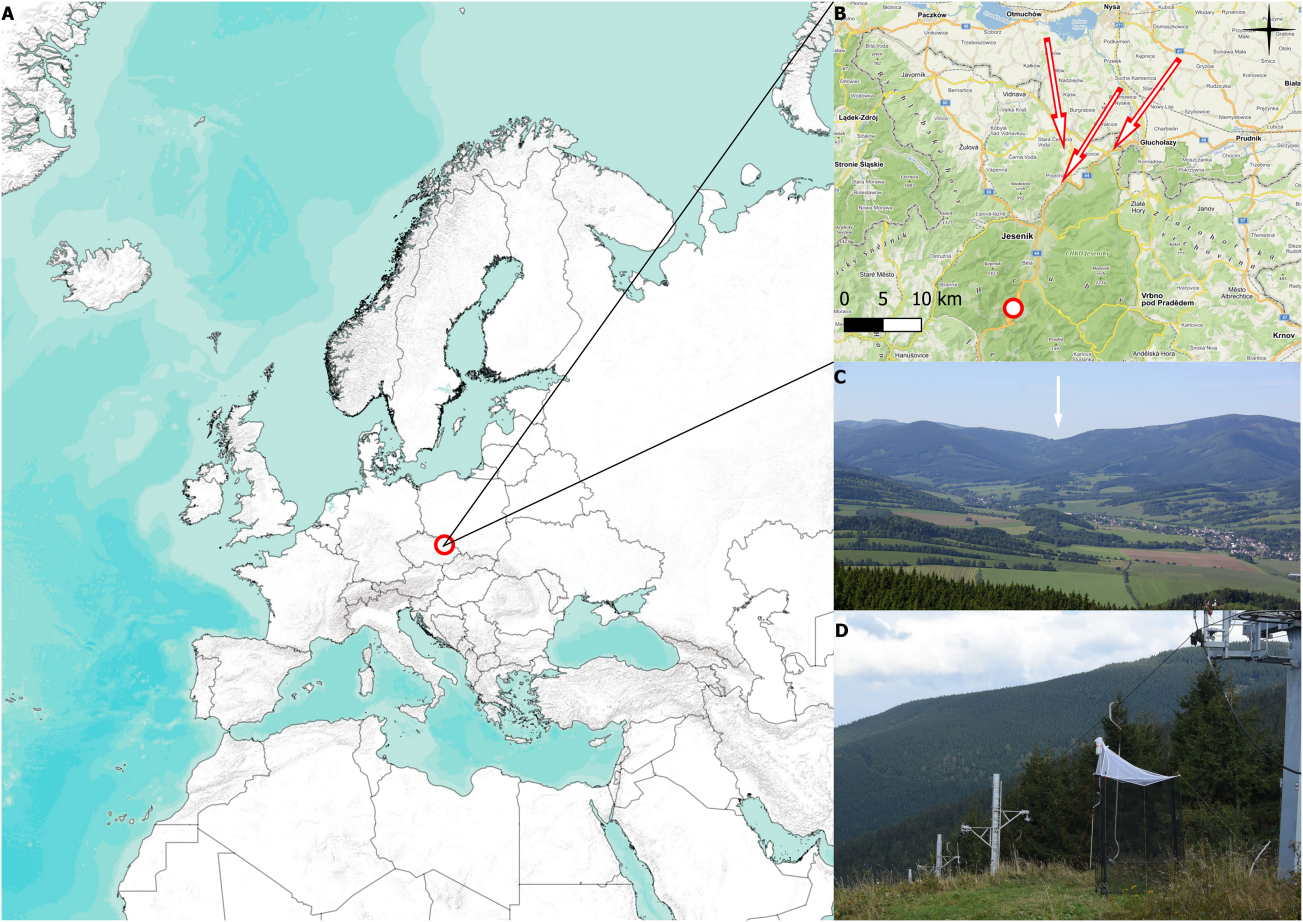
Collecting site and traps. (A) Position of the mountain pass Červenohorské sedlo, Czech Republic. (B) Detailed map of the Jeseníky mountains, where the red dot indicates placement of Malaise trap, the red arrows represent presumed migratory roads. (C) View of Červenohorské sedlo from the Polish side. Červenohorské sedlo is pointed out with white arrow (photo by Martin Vavřík). (D) Photograph of Červenohorské sedlo and two one-side-blocked Malaise trap (photo by Jiří Hadrava). The map was created using QGIS software 3.14. (2022).

Our study was carried out at the Červenohorské sedlo mountain pass in Jeseníky Mountains, NE of the Czech Republic, 50.1245331N, 17.1537733E, 1,020 meters above sea level (m a.s.l.) (see Figs. 1A and 1B). Červenohorské sedlo is a deep break that divides the Jeseníky mountains into the western and eastern ridge, generating an ideal path for aerial migration of insects, birds, and bats moving in the north-south direction (Vavřík et al., 2016), see Fig. 1C.

Two Malaise traps were placed at the top of a ski slope (1,020 m a.s.l.), rising from the river dale (850 m a.s.l.), which provides an ideal and shortest fly path for migratory flying animals to cross the mountains in the lowest possible elevation. We used two one-side blocked Malaise traps to distinguish between southward and northward migration, see Fig. 1D. The traps were operated from 2 August to 18 October in 2018, from 4 August to 31 October in 2019, from 2 August to 2 October in 2020, and from 27 September to 28 October in 2021, according to the annual weather conditions. We emptied the traps daily, approximately at 1900 (UTC+1). Data from the pilot project conducted in 2018, when the placement of the trap was adjusted, and data from the year 2021, lacking August and September observations, are displayed; however, they were omitted from the analysis of the sex ratio, species ratio, and weather conditions on migration.

To explore the effect of Malaise trap size and proportion of the Malaise traps, various traps were used from 2018 to 2020 and in 2021: from 2018 to 2020, smaller traps were used (lower side height = 1 m, higher site height = 2 m, wide = 1.3 m), and in 2021, higher traps (lower side height = 2 m, higher site height = 2.5 m, wide = 1.3 m ) were used in order to test the effect of trap diameter and shape on its effectivity. Data from 2019 and 2020 were collected by the same traps and they were used for quantitative analysis of inter-annual differences in hoverfly abundance.

Weather data (temperature (°C), wind speed (m/s), wind direction, precipitation (mm/day), and relative barometric pressure (hPa)) were retrieved from Hadex WH 1080 weather station, placed in the close proximity (<100m) of the traps.

### Determination and statistics

All specimens were pinned and are deposited in the private collections of the authors. The species were identified using keys: Hippa, Nielsen & Steenis (2001), van Veen (2010), Láska, Mazánek & Bicík (2013), Bot & van de Meutter (2020). In genus *Sphaerophoria*, females were not identified, but they were assumed to be *Sphaerophoria scripta* (L., 1758), as no other species of the genus was recorded within males.

Statistical analysis and data visualization were performed in R software, version 4.1.2 (R Core Team, 2021). We used a generalized linear model (GLM) assuming a quasibinomial distribution of errors, in order to assess sex ratio discrepancy between years and species, where only data from peaks of migration (PM–period, when more than 95% of individuals per day were caught by north-facing trap and was preceded by at least one day without any observations, to avoid the bias from local populations), were used (2019: 13 to 29 October; 2020: 3 September to 2 October). Significance at the 0.05 level was determined by ANOVA. Due to the extremity of the *p*-values, *post-hoc* corrections were not necessary. The analysis of species phenology was performed by fitting local polynomial regression (Cleveland, Grosse & Shyu, 1992), α = 0.5. Shannon & Weaver (1949) diversity index was used to compare species diversity between years. To assess influence of weather, we used Canoco software v. 5.0 (Ter Braak & Šmilauer, 2012), to perform partial RDA analysis, with stepwise selection of variables, where year was included as random factor.

## Results

**Figure 2:**
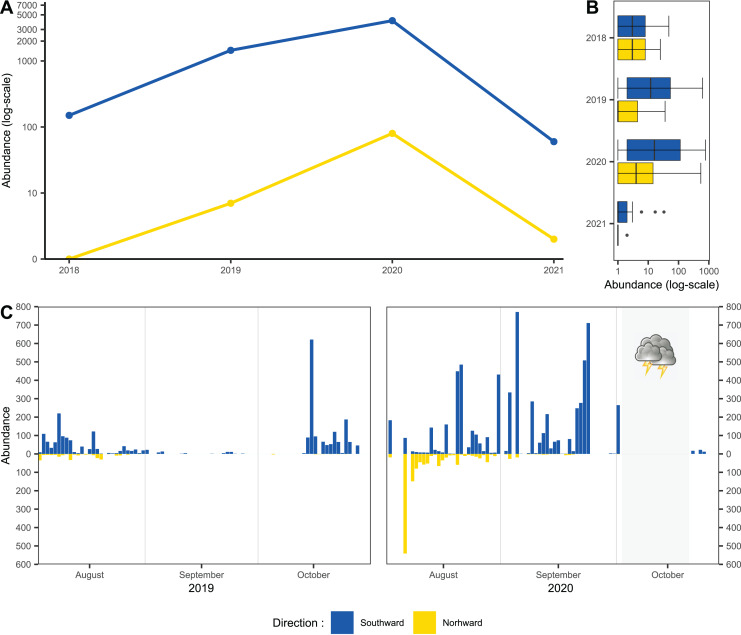
Total abundance of hoveflies migrating southward. (A) Total abundance during the pinnacles of migration, logarithmic scale of the y-axis. (B) Box and whiskers plot, with median of abundance of species during the entire season in all monitored years, log scaled, (C) Numbers of specimens caught flying northward or southward in 2019 and 2020. The blue colour represents specimens flying southward, the yellow colour represents specimens flying northward. The grey area represents the period of the tempest.

Usage of one-side-blocked Malaise traps allowed us to distinguish individuals migrating southward, from local populations. In early August, the numbers of specimens caught in the north and south traps were almost similar (2 August to 20 August 2020: 1,155 flying northward and 1,117 flying southward). During September and October, we recorded specimens only in the trap catching hoverflies migrating southward; see Fig. 2C. In total, we recorded 11,530 hoverflies, where 9,681 specimens were caught flying southward; see Fig. 2A. During the peaks of migration (27 September to 18 October in 2018; 13 to 29 October in 2019; 3 September to 2 October in 2020; 27 September to 29 October in 2021), we recorded 5,743 specimens flying southward and only 89 flying northwards; see Fig. 2B.

### Species diversity

Overall, 31 species of hoverflies were recorded flying southward during the peaks of migration (Table 1). Only four of them made up 81.51% of migrants. The most abundant species was *Melanostoma mellinum* (L., 1758) (40.26%), followed by *Episyrphus balteatus* (19.64%), *Sphaerophoria scripta* (14.66%), and *Eupeodes corollae* (6.95%), see Table 1. According to Shannon-Weaver index, species diversity differed between years, with highest diversity in 2020 (H = 1.947) and lowest in 2021 (H = 0.178), see Table 1.

**Table 1:** Diversity of migrating hoverflies and their abundance.

Species	2018			2019		2020		2021		Total abundance		Migratory status
*Cheilosia canicularis* (Panzer, 1801)						17/2				17		N
*Dasysyrphus* sp. Enderlein, 1938	2					8				10		
*Didea alneti* (Fallén, 1817)						2				2		W
*Didea fasciata* Macquart, 1834						4/1				4		W
*Episyrphus balteatus* (De Geer, 1776)	39			550/4		534/2		5		1,128		S
*Eristalis arbustorum* (L., 1758)						18				18		S
*Eristalis nemorum* (Poda, 1761)						4				4		W
*Eristalis pertinax* (Scopoli, 1763)						25				25		W
*Eristalis tenax* (L., 1758)	7			1		123/2		10		141		W
*Eupeodes corollae* (F., 1794)	7			223		165/3		4		399		S
*Eupeodes latifasciatus* (Macquart, 1829)				1		2				3		W
*Eupeodes lundbecki* (Soot Ryen, 1946)						9				9		S
*Eupeodes luniger* (Meigen, 1822)				1		17				18		S
*Helophilus pendulus* (L., 1758)						58/1				58		W
*Helophilus trivittatus* (F., 1805)						17/1		1		18		W
*Lapposyrphus lapponicus* (Zetterstedt,1838)				1		14				15		W
*Megasyrphus erraticus* (L., 1758)						2				2		W
*Meliscaeva auricollis* (Meigen, 1822)						22				22		W
*Meliscaeva cinctella* (Zetterstedt, 1843)								1		1		W
*Melanostoma mellinum* (L., 1758)	43/1			443/1		1,798/12		28/1		2,312		S
*Myathropa florea* (L., 1758)						18				18		N
*Parasyrphus lineolus* (Zetterstedt, 1843)						11/2				11		W
*Platycheirus albimanus* (F., 1758)	5					259/31				264		*
*Scaeva pyrastri* (L., 1758)	9			4		20				33		S
*Scaeva selenitica* (Meigen, 1822)	7			1		10		5		23		S
*Sphaerophoria* cf. *scripta* (L., 1758)	7			204/2		630/8		1		842		S
*Syrphus ribesii* (L., 1758)	1					29		1		31		W
*Syrphus torvus* Osten Sacken, 1875	18					174/8		2		194		*
*Syrphus vitripennis* Meigen, 1822				7		43				50		S
*Xanthandrus comtus* (Harris, 1780)	4			8		7		1		20		W
*Xylota sylvarum* (L., 1758)						1				1		W
Other**				14		36/7				50		
Summary	149/1			1,458/7		4,077/80		59/1		5,743/89		
Shannon-Weaver diversity index	0.389			1.281		1.947		0.178				

**Notes:**

* Not treated (based on Speight, 2020).

** Other: damaged specimen, impossible to identify.

Species recorded flying southward (left side of the slash) and northward (right side of the slash) and their abundances during the pinnacles of migration (27.9-18.10.2018.; 13.-29.10.2019; 3.9.-2.10.2020; 27.9.-29.10.2021), their total abundance and migratory status: S, strongly migratory; W, weakly migratory; N, most likely nonmigratory species, recorded due to the phenology of species.

### Timing of migration

**Figure 3:**
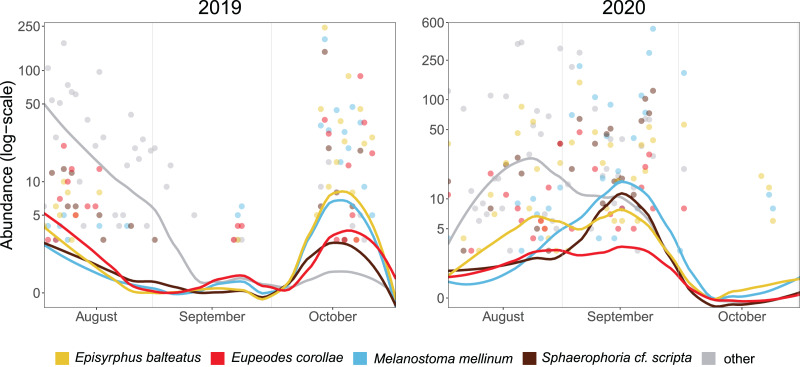
Phenology of four most abundant migrants. Abundance of the four most common migrants in 2019 and 2020. Lines represent local polynomial regression, α = 0.5.Y-axis log-scaled.

The median of PM differed between years (21 October in 2019, 17 September in 2020). Besides the overall timing, even species differed in the phenology of migration. In 2019, the abundance of *Episyrphus balteatus*, *Melanostoma mellinum*, and *Sphaerophoria scripta* peaked on 14 October, whereas *Eupeodes corollae* was most abundant on 23 October. In 2020, migration of most species started almost a month earlier than in the 2019, and the PM of species differed. *Eupeodes corollae* and *Episyrphus balteatus* appeared first (5 September) and were consistently abundant during the migration period. *Sphaerophoria scripta* and *Melanostoma mellinum* were observed later, with peak of abundance on 24 September (see Fig. 3).

**Figure 4:**
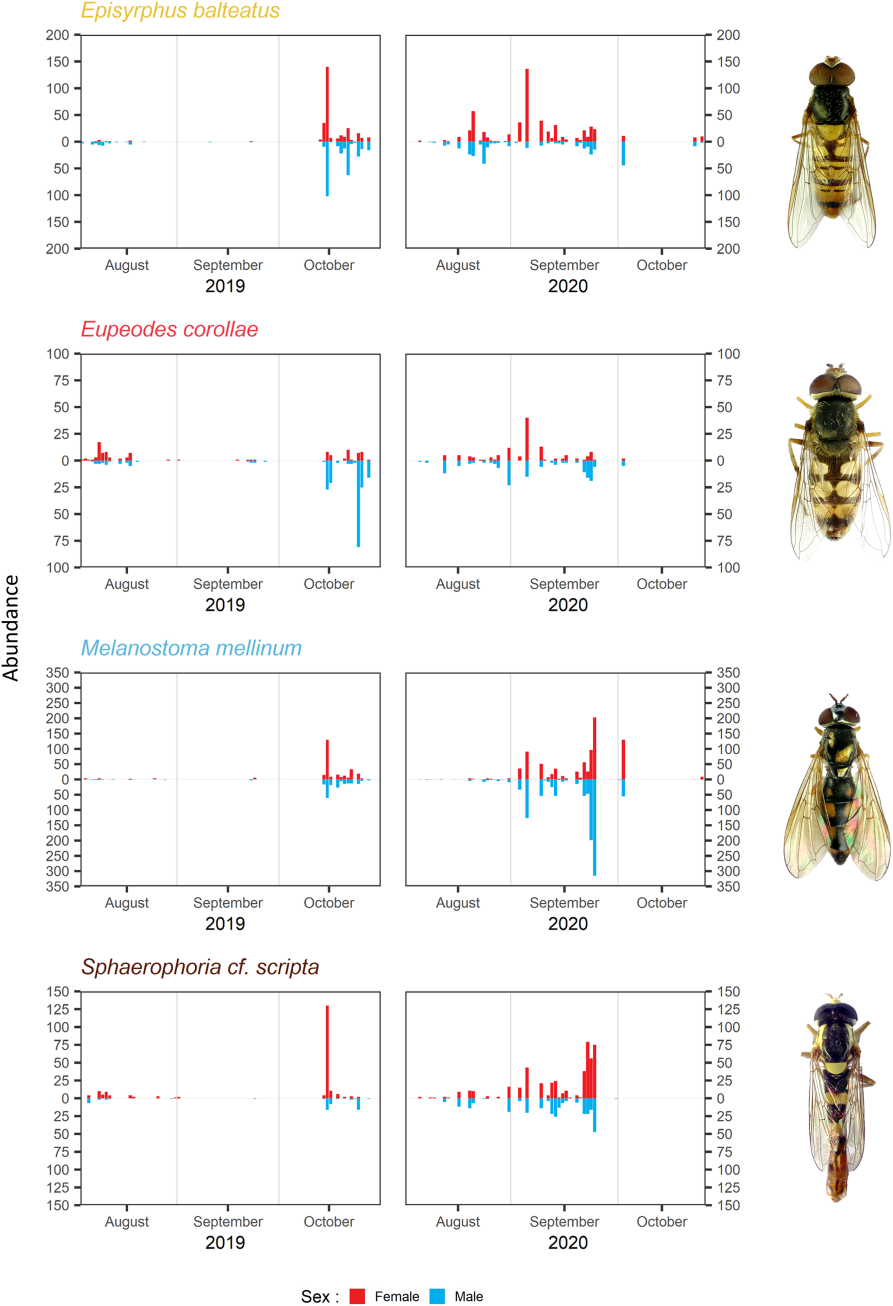
Total abundance and sex ratio of four most common migratory species. Total abundance of the four most common migratory species and their sex ratio in 2019 and 2020. Photos of the species were taken by the authors.

The timing of migration differs for males and females. The PM was similar for both sexes during both years in *Melanostoma mellinum*. In *Eupeodes corollae*, males peaked in 2019 at the end of the migration period, when females were consistently abundant. On the contrary, in 2020, the peak of *Eupeodes corollae* female migration was observed at the beginning of the migration period, when males were consistently abundant with small peak at the end of the migration period. However, in both years, on average, females migrated earlier than males in *E. corollae*. The abundance peak in *Episyrphus balteatus* was similar for both sexes in 2019. In 2020, females of *Episyrphus balteatus* peaked at the beginning of the migration period, followed by males later on. In *Sphaerophoria scripta*, the peak of female migration in 2019 was at the beginning of the migration period, where males were consistently abundant. In 2020, both sexes of *Sphaerophoria scripta* migrated at the same time; see Fig. 4.

### Sex ratio of migrants

The sex ratio of species varied between years. Considering the overall sex ratio, males and females did not differ (2019: 51.75% F, 48.25% ; 2020: 54.82% F, 45.18% M; binomial GLM, F-test, *p* = 0.4771, Df = 1, *R*^2^_L_ = 0.003, nM = 2,156, nF = 2,391). However, the sex ratio of migrants differed between species (binomial GLM, F-test, *p* = 2.365e−06, Df = 3, *R*^2^_L_ = 0.205) and there was a statistically significant interaction between species and year (binomial GLM, F-test, *p* = 6.421e−05, Df = 3, *R*^2^_L_ = 0.15); see Table 2.

**Table 2:** ANOVA for quasibinomial GLM on sex ratio and tis fluctuations.

	df	deviance	*p* value	*R* ^2^_L_
Season	1	2.925	0.4771	0.003
Species	3	190.730	2.365e−06	0.205
Season * Species	3	140.492	6.421e−05	0.15

**Note:**

Results of GLM assuming quasibinomial distribution of errors, link function: logit. The statistical significance of the variables was based on the F-ratio test. Null deviance: 931.16. Residual deviance: 597.01. Residual degrees of freedom: 102, *R*^2^_L_: Pseudo-R-squared.

**Figure 5:**
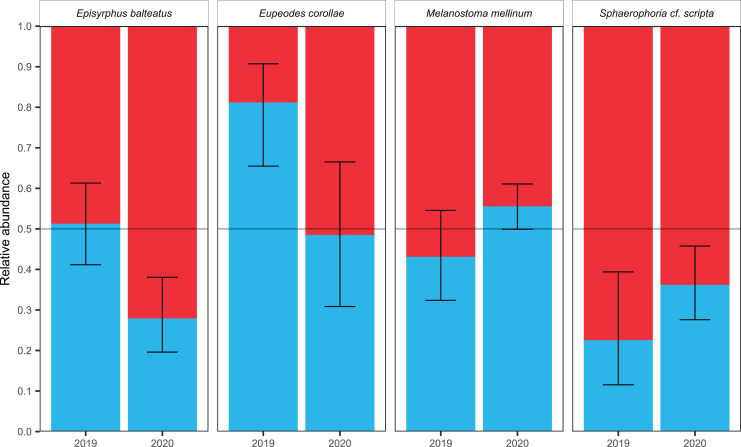
Sex ratio of four most common migrants and its fluctuation. Sex ratio of the four most common migrants in 2019 and 2020. The 95% confidence interval of the binomial distribution is plotted, regarding to GLM. The red colour represents females, the blue colour represents males.

Female-biased sex ratio was observed in *Sphaerophoria scripta* during both years; see Table 3. The male-biased sex ratio was observed in *Eupeodes corollae* in 2019; however, in 2020, the sex ratio was insignificantly female-skewed; see Table 3. In *Episyrphus balteatus*, the sex ratio was even in 2019; but females prevailed in 2020; see Table 3. The sex ratio of *Melanostoma mellinum* was shifted toward the females in 2019, and toward the males in 2020; however, in both years, observed trend was insignificant; see Table 3. For the graphical comparison of the sex ratio of species, see Fig. 5.

**Table 3:** Sex ratio of migrants.

Species		Year	Females (%)	Males (%)	LCI	UCI	*n*
*Episyrphus balteatus*		2019	48.73	51.27	0.412	0.613	550
*Episyrphus balteatus*		2020	72.1	27.9	0.196	0.381	534
*Eupeodes corollae*		2019	18.83	81.17	0.655	0.907	223
*Eupeodes corollae*		2020	51.52	48.48	0.308	0.665	165
*Melanostoma mellinum*		2019	56.88	43.12	0.324	0.545	443
*Melanostoma mellinum*		2020	43.17	56.83	0.499	0.611	1,798
*Sphaerophoria* cf. *scripta*		2019	77.45	22.55	0.115	0.394	204
*Sphaerophoria* cf. *scripta*		2020	63.8	36.2	0.276	0.458	630

**Note:**

Sex ratio of four most common species based on GLM, assuming quasibinomial distribution of errors, link function: logit. LCI, lower 95% confidence interval; UCI, upper 95% confidence interval; *n*, number of specimens.

### Weather conditions

**Figure 6:**
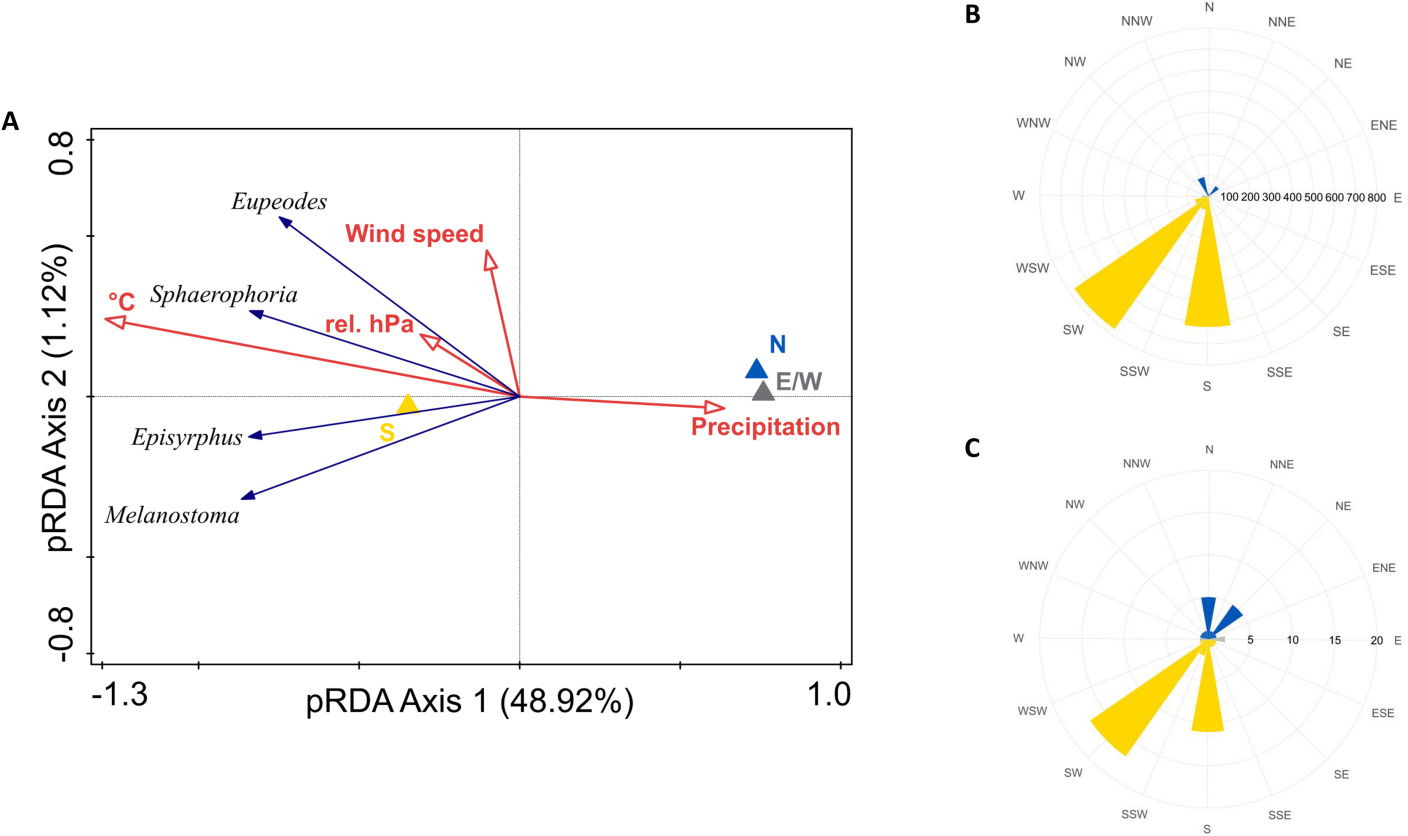
Weather and its effect on hoveflies migration. (A) Partial RDA analysis of weather conditions and their effect on hoverfly migration. N, northerlies; S, southerlies; E/W, eastern, western winds; °C, average daily temperature; rel. hPa, relative barometric pressure. (B) Hoverflies captured during southward migration peaks in 2019 and 2020 respectively to the daily average wind direction (northern–yellow, southern–blue, eastern/western–grey). (C) Daily average wind direction during the migration peaks in 2019 and 2020. Northward–yellow, southward–blue, east/west winds–grey.

Intensity of migration of all species was positively influenced by southerly winds, temperature, and relative barometric pressure, and negatively by precipitation. Wind speed marginally influenced migration of *Eupeodes corollae* and *Sphaerophoria scripta*, see Fig. 6A. The partial RDA provided quantification of proportion of variance, explained by weather conditions. Partial RDA axis one (eigenvalue = 0.4892) and axis two (eigenvalue = 0.0112) explained 51.04% of variation in species data. Weather conditions explained 51.8% of variation in data. Monte Carlo permutation test was performed on all constrained axes (pseudo-F = 7, *p* = 0.0001, number of permutations = 10,000). Highest abundance of migrants was observed during the southern wind, see Fig. 6B; however, the wind on the Červenohorské sedlo was rarely northerly, see Fig. 6C.

## Discussion

### Species diversity

From 53 hoverflies reported as migratory by Speight (2020), we recorded 29 species. Consistently with previous studies, we found that *Platycheirus albimanus* (F., 1758) and *Syrphus torvus* Osten Sacken, 1875 are migratory (Aubert, Aubert & Goelding, 1976; Jensen, 2001; Gatter et al., 2020; Martens, 2020), even though they were not listed as migratory by Speight (2020). Two species, *Cheilosia canicularis* (Panzer, 1801) and *Myathropa florea* (L., 1758), were recorded in a single year, 2021, and due to the lack of any other observations, it will be too early to conclude them as migratory. The composition of the migrants differed between all monitored years. This pattern could be driven by the specificity of each year given *e.g.*, by climate variation and weather conditions which may affect the length of development or survival of particular species as well as the timing and intensity of migration on both local and large-scale spatial level.

### Timing of migration

The timing of migration of the four most abundant migrants differed between species and years. Timing of migration is a complex phenomenon, believed to be influenced by the circadian clock (Denlinger et al., 2017; Zhan et al., 2011), weather conditions (Drake, Drake & Gatehouse, 1995; Knoblauch, Thoma & Menz, 2021), or co-migration with predators (Cohen & Satterfield, 2020). However, none of the presented studies explain the discrepancy between years and species. Promising theory, proposed for birds, seemed to be ‘body size theory’ (Ketterson & Nolan, 1976), suggesting that larger individuals are ‘better suited to survive the colder and less predictable climates at higher latitudes’ (Nebel, 2007). This theory is perfectly suitable for endotherms (explaining even a sex ratio discrepancy). However, the observed migration patterns of hoverflies directly contradict body size theory, with smaller species (*Melanostoma mellinum*, *Sphaerophoria scripta*) migrating later or simultaneously with larger ones (*Episyrphus balteatus*, *Eupeodes corollae*). Moreover, Knoblauch, Thoma & Menz (2021) observed the same pattern in dragonflies, when the median day of passage came later for smaller species. We hypothesize that the timing of migration may be influenced, in addition to weather and overall seasonal conditions, by a (1) dispersion capacity, where larger species have bigger wing area and higher wing aspect ratio, so they arrive earlier, (2) a minimal temperature threshold for species, where smaller, purely ectothermic, species are able to withstand harsh conditions. This study provides a first step towards a better understanding of the phenology of migration among broader taxa; however, confirmations of our finding require further research, where work is currently underway to determine the temperature threshold and flight capability of migrants.

We observed differences in phenology between males and females. In the four most abundant species, the peak of female abundance occurred earlier than the migration peak of males, with the exception of *Melanostoma mellinum*, where the peak of migration occurred at the same time for both sexes. Earlier migration of females is believed to be influenced by their earlier emergence, in order to search for suitable oviposition habitat (Guo et al., 2019). However, earlier migration of females was observed even in *Episyrphus balteatus*, which overwinter mainly as adults (Speight, 2020). Furthermore, in *Melanostoma mellinum*, which overwinter as larvae (Speight, 2020), the females migrated at the same time as the males. We cannot rule out the theory of oviposition in *Eupeodes corollae* or *Sphaerophoria scripta*, which overwinter as larvae (Speight, 2020). On the basis of our results, we challenge the theory of oviposition as a general explanation for earlier female migration. It is very likely that the earlier appearance is driven by population dynamics or higher flight capability of females (Cui et al., 2016; Guo et al., 2019).

### Sex ratio of migrants

The sex ratio of the four most common migrants differed between years. Previous studies suggested various explanations for the sex ratio bias in migrating swarms: methodological bias, driven by placement of traps or various migration routes (Nielsen, 1968; Svensson & Janzon, 1984; Steinbauer, 2003), differential mortality of sexes (Tabadkani et al., 2013), various flight and dispersion capabilities (Steinbauer, 2003; Guo et al., 2019), or fat reserves allowing to survive harsh conditions of higher latitudes for longer time (Steinbauer, 2003; Brattström et al., 2018; Guo et al., 2019). Given the same placement of traps in our study, our results do not support hypothesis explaining biased sex ratios in the light of spatial heterogeneity in the distribution of migrating insect (Nielsen, 1968; Svensson & Janzon, 1984). The significant variation of the sex ratio between years challenges the theory of higher flight and dispersion capability of one of the sexes (Guo et al., 2019). However, most studies dealing with the sex ratio discrepancy were not based on long-term observations. At this point, we cannot fully resolve this question, and further research is needed. A tentative explanation is that the sex ratio may be altered on population level by various factors, such as interannual variation in weather or parasitism (Davis & Rendón-Salinas, 2010).

### Weather conditions

Intensity of migration was greatly affected by weather. Hoverfly migration was positively related to the southerlies (headwind for migrating hoverflies), temperature, and barometric pressure and negatively by rainfall. We assume that highest abundance of migrants in headwind conditions is largely affected by methodology of sampling. While masses of migrating insects keep high above the ground, when flying with tailwinds southward, thus being hardly detectable by Malaise traps, they migrate at the ground-level at so-called flight boundary layer (Taylor, 1974), to reduce energy costs and increase flight efficiency, when facing headwinds (Gao et al., 2020), hence they are easily detectable by Malaise traps. However, southerlies are strongly predominant on the study site, so the testing of possible selection for northern tailwinds is further limited. Moreover, autumnal migrants are strongly limited in the selection of wind direction, as the weather in the north is gradually becoming unbearable for them (Gao et al., 2020). The observed correlation between actual weather conditions and detected migration could be either driven by direct effect of weather on migration behaviour (Hu et al., 2016), or by constrains of methodology used in our study.

### Malaise trap as method for migration research

We have demonstrated the functionality of two single-sided Malaise traps for the study of hoverfly migration at ground-level. One-side-blocked Malaise trap represents a cost-effective method, helping to understand the natural history of migrants. The method of two single-sided Malaise allowed us to distinguish between migratory and resident individuals and, in addition, to determine the start of migration according to the proportion of individuals flying southward and northward, contrasting with studies using classical Malaise-like traps such as those used by Aubert, Aubert & Goelding (1976) or Gatter et al. (2020). On the contrary, Malaise traps are known to be selective (Campbell & Hanula, 2007) and placement sensitive (Malaise, 1937). Furthermore, some hoverflies are known to evade the Malaise traps (Burgio & Sommaggio, 2007), as we observed where for example individuals of the *Eristalis tenax* (L., 1758) dodged or rammed and escaped the traps. Our design could be improved by combining the Malaise trap with window traps or Möricke traps.

## Conclusions

Four years of observation of the autumn migration of hoverflies revealed a relationship between body size and timing of the migration, where bigger species migrate earlier than smaller ones. This phenomenon, previously observed only in dragonflies, is strongly counterintuitive, moreover in ectotherms. In addition, the sex ratio of migrants differs from 1:1 and is species-specific and year-specific. These results point the way to the better understanding temporal and spatial fluctuation of migratory insects. Future studies should focus on the drivers of the sex asymmetric migration and phenological heterogeneity of insect migration.

## Supplemental Information

10.7717/peerj.14393/supp-1 Supplemental Information 1 Sex ratio of four most common migrants.

10.7717/peerj.14393/supp-2 Supplemental Information 2 Data on diversity of migrants. Data used for Shannon index computation.

10.7717/peerj.14393/supp-3 Supplemental Information 3 R code for sex ratio and diversity analysis.

10.7717/peerj.14393/supp-4 Supplemental Information 4 Data on weather during the pinnacles of migration.

## Additional Information and Declarations

### Data Availability

The following information was supplied regarding data availability: The code and raw data are available in the Supplemental Files.
